# Myelodysplastic syndrome in an infant with constitutional pure duplication 1q41-qter

**DOI:** 10.1038/s41439-018-0008-8

**Published:** 2018-05-21

**Authors:** Hirokazu Morokawa, Motoko Kamiya, Keiko Wakui, Mikiko Kobayashi, Takashi Kurata, Kazuyuki Matsuda, Rie Kawamura, Hiroyuki Kanno, Yoshimitsu Fukushima, Yozo Nakazawa, Tomoki Kosho

**Affiliations:** 1 Department of Pediatrics, Shinshu University School of Medicine, Matsumoto, Japan; 2 Center for Medical Genetics, Shinshu University Hospital, Matsumoto, Japan; 3 Department of Medical Genetics, Shinshu University School of Medicine, Matsumoto, Japan; 4 Department of Pathology, Shinshu University School of Medicine, Matsumoto, Japan; 5 Department of Laboratory Medicine, Shinshu University Hospital, Matsumoto, Japan

## Abstract

We report on a Japanese female infant as the fourth patient with the constitutional pure duplication 1q41-qter confirmed by chromosomal microarray and as the first who developed myelodysplastic syndrome (MDS) among those with the constitutional 1q duplication. Common clinical features of the constitutional pure duplication 1q41-qter include developmental delay, craniofacial characteristics, foot malformation, hypertrichosis, and respiratory insufficiency. The association between MDS and the duplication of the genes in the 1q41-qter region remains unknown.

The constitutional pure duplication 1q41-qter is a condition with an excess of the 1q41-qter region but without imbalances of other chromosomal regions, associated with chromosomal rearrangements^1–3^. In only three among eight non-mosaic cases of pure duplication 1q41-qter published to date, imbalances of other chromosomal regions were excluded through molecular cytogenetic techniques, including chromosomal microarray (Table 1)^2, 3^. We report the fourth case of constitutional pure duplication 1q41-qter confirmed by chromosomal microarray and the first patient with a constitutional 1q duplication who developed myelodysplastic syndrome (MDS).

**Table 1:** Patients diagnosed with pure duplication 1q41-qter with chromosome microarray

Reference	Shin et al. [2]	Watanabe et al. [3]		Present patient
		Case 1	Case 2	
Age at examination/sex	6 mo/M	1 y	14 y	1 y 7 mo/F
Cytogenetic analysis	G-band, FISH, CMA	G-band, CMA	G-band, CMA	G-band, FISH, CMA
G-banded karyotype	46,XY,der(11)t(1;11) (q41;p15.5)	46,XY,der(15)t(1;15) (q41;p?)	46,XY,der(15)t(1;15) (q41;p11.2)	46,XX,der(7)t(1;7)(q41;p22.3)
Duplication size (Mb)		26.8	32.6	26.7
Origin	Mat			De novo
Birth information				
Gestational weeks	39			37
Weight (g)	2820			2444
OFC (cm)				34.5
Postnatal growth impairment	+			−
Developmental delay	+	+	+	+
Intellectual disability	+	+	+	
Craniofacial features				
Macrocephaly	+	+	−	+
Large fontanels	−	−	−	+
Prominent forehead	+	+	+	+
Widely spaced sutures				+
Epicanthic folds				+
Hypertelorism		+	−	+
Triangle face	+	−	+	+
Downslanting palpebral fissures	+	−	+	+
Broad nasal bridge	+	+	+	+
High palate		−	−	+
Micro/retrognathia	+	+	+	+
Low-set ears	+	+	+	+
Short neck		+	+	+
Widely spaced nipples				+
Hand/foot malformation		−	+ (Overlapping toes)	+ (Syndactyly)
Hypertrichosis		+	−	+
CNS abnormalities		−	+ (Ventriculer dilatation)	−
Cardiac malformations	−	−	−	−
Urogenital anormalies		−	+	−
Respiratory insufficency		−	+ (Recurrent infection)	+
Gastrointestinal abnormalities	−	−	−	
Others				MDS

*CMA* chromosomal microarray, *CNS* central nervous system, *F* female, *M* male, *Mat* maternal, *MDS* myelodysplastic syndrome, *mo* month(s), *OFC* occipitofrontal circumference, *y* year(s)

The proband was a girl, born as the first child of healthy nonconsanguineous Japanese parents. She was delivered by cesarean section at 37 weeks and 0 days of gestation after an uneventful pregnancy. Her birth weight was 2444 g (−0.3 SD), length 46.0 cm (−0.6 SD), and occipitofrontal circumference 34.5 cm (+1.4 SD). Apgar scores were 4 at 1 min and 7 at 5 min. She developed respiratory distress and received mechanical ventilation for 2 days, followed by oxygen supplementation for 3 months. Her physical findings at age 1 month included craniofacial features (macrocephaly, a large and prominent forehead, epicanthic folds, hypertelorism, a depressed nasal bridge, a high and narrow palate, retro/micrognathia), hypertrichosis, widely spaced nipples, a right single palmar crease, and syndactyly of the first to third toes of both feet. Brain, cardiac, and abdominal ultrasonography showed no abnormalities.

At age 1 month, her white blood cell count was 11.2 × 10^3^/µL with leukemic blasts of 6.0% (Fig. 1a). The hemoglobin level was 7.1 g/dL, and the platelet count was 55 × 10^3^/µL. She was transferred to our hospital. The initial bone marrow aspiration was performed at age 6 weeks. The smear showed blast cells (Fig. 1b). The clot section showed age-appropriate hypercellular marrow (Fig. 1c). Megakaryocytes were increased in number, and many of them displayed dysplasia, such as abnormally separated nuclear lobes (Fig. 1c, d). Erythroblasts exhibited diffuse distribution, and no apparent erythroblastic islands were found (Fig. 1d). Immunohistochemical examination revealed CD41-positive micromegakaryocytes and small megakaryocytes (Fig. 1e). The follow-up bone marrow aspiration was performed at the age of 3 months. The clot histology showed a mild increase in blast cells in addition to the findings in the initial examination (data not shown). Immunohistochemistry showed scattered p53-positive cells and slightly increased CD34-positive blasts. There was fetal hemoglobin expression in some erythroblasts and an increase in CD42b-positive megakaryocytes, which were small and mononuclear (data not shown).

**Fig. 1:**
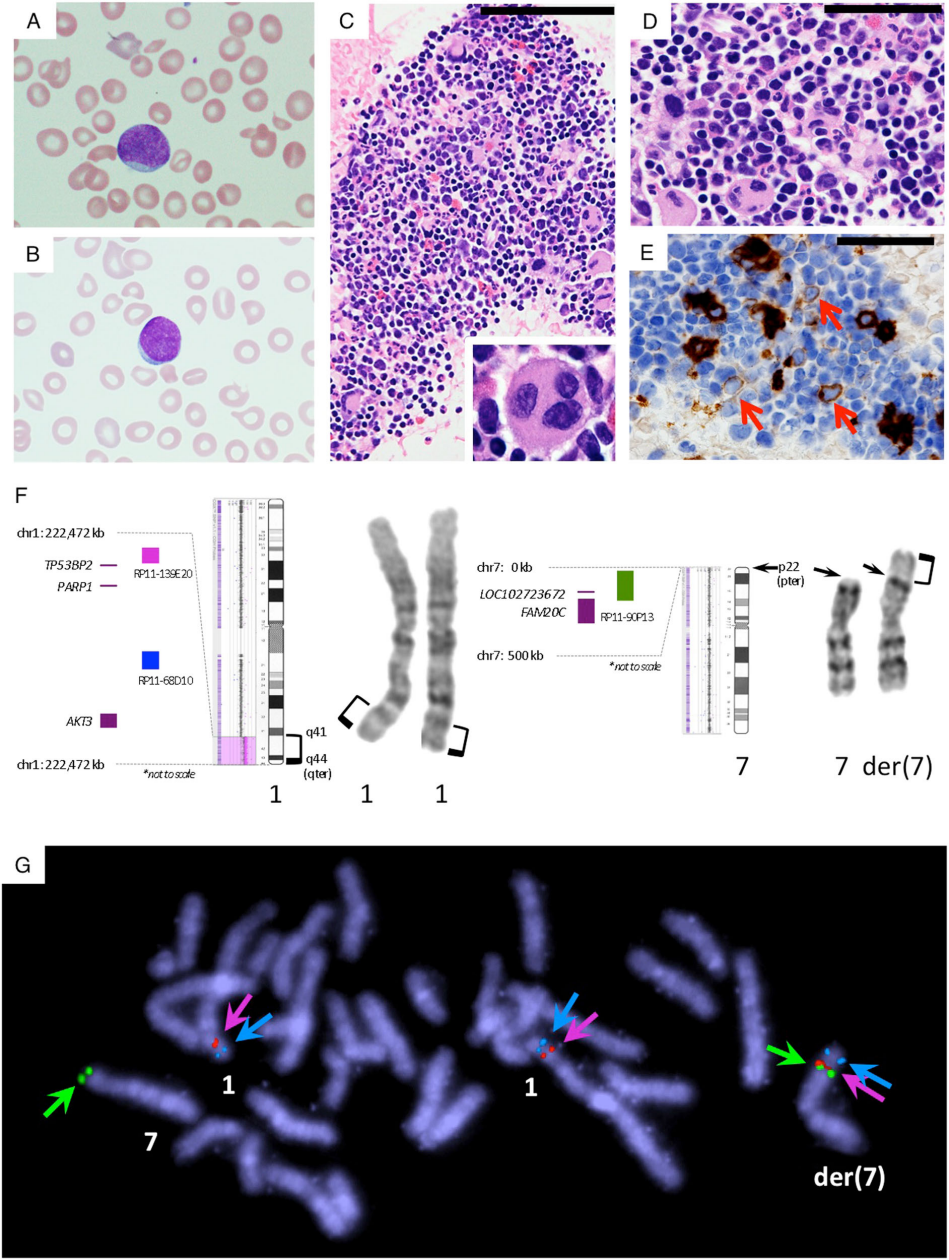
Hematological and molecular cytogenetic findings. **a** A blast cell in the peripheral blood smear at age 1 month. **b**: A blast cell in the bone marrow smear at age 6 weeks. **c**, **d** Histopathology and immunohistochemistry for CD41 of the clot section of bone marrow aspirate. **c** Age-appropriate hypercellular marrow is shown. Megakaryocytes are increased in number and display dysplasia with abnormally separated nuclear lobes (inset) (hematoxylin and eosin staining, original magnification, x400; scale bar, 100 μm, inset; original magnification, x1000). **d** Erythroblasts are distributed diffusely, and no erythroblastic islands are present (hematoxylin and eosin staining, original magnification, x1000; scale bar, 50 μm). **e** CD41-positive micromegakaryocytes and small megakaryocytes (arrows) are observed (immunostaining for CD41; original magnification, ×1000; scale bar, 50 μm). **f**, **g** The results of conventional and molecular cytogenetic analyses using peripheral blood at age 2 months. **f** Partial G-banded karyotype and chromosome microarray analysis of chromosomes 1 and 7. The 7pter breakpoint of derivative chromosome 7 is indicated by arrows. A 26.74-Mb duplicated region of 1q41-qter (chr1:222,472,360–249,208,146) is indicated as a pink shaded background in the microarray plot and as the brackets. No apparent copy number loss was observed at 7p22.3-pter by microarray. **g** Result of metaphase FISH using three kinds of probes: red: RP11-139E20 (1q41, chr1:223,035,115–223,191,269), aqua: RP11-68D10 (1q42.3, chr1:236,274,313–236,474,743), and green: RP11-90P13 (7p22.3, chr7: 23,874–203,581 *including the region of the most distal probe of chromosome 7 (chr7: 41,243–41,291) on our microarray). Green signals for the subtelomere 7p probe (RP11-90P13) are retained on the der(7) as well as on the normal chromosome 7

G-banded karyotyping of the bone marrow cells to detect the chromosomal abnormalities responsible for hematological malignancy showed 46,XX,add(7)(p22) at age 6 weeks. This chromosomal aberration was thought to be constitutional. G-banding of phytohemagglutinin-stimulated peripheral blood was performed at age 2 months when the peripheral blood blasts decreased to 2.0%, and the karyotype showed the same abnormalities on chromosome 7 (Fig. 1f). Parental karyotypes were normal. The follow-up G-banding of the bone marrow cells at age 9 months showed trisomy 8 as an additional chromosomal aberration in 5/22 (22.7%) cells with 2.0% blasts.

The peripheral blood showed a persistently low level of monocytes (>1 × 10^9^/L was not maintained), hemoglobin (<10 g/dL), and platelet (<10 × 10^4^ /µL). Leukemic blasts were detected in 2–4% of the bone marrow cells and in 3–9% (1–2 months of age) and ~4% (~3 months of age) of the peripheral blood cells. Auer rods were not found in the peripheral blood smear or bone marrow smear. In view of all these hematological findings as well as the exclusion of infectious diseases, metabolic disorders, or other causes of cytopenia or dysplasia, she was diagnosed with refractory anemia with excess blasts-1 (RAEB-1) according to the 2008 revision of the World Health Organization (WHO) classification^4^ and with MDS with excess blasts-1 (MDS-EB-1) according to the 2016 revision of the WHO classification^5^.

At age 9 months, her hemoglobin level and platelet count increased to 12 g/dL and 70 × 10^3^/µL, respectively, with no change in the peripheral blood blast percentage. However, her platelet count gradually decreased to approximately 10 × 10^3^ /µL at age 1 year and 4 months, requiring platelet transfusions every 2 weeks thereafter. Her hemoglobin level also gradually decreased to 6 g/dL at age 1 year and 6 months. She underwent bone marrow transplantation from her HLA-matched mother at age 1 year and 7 months and achieved complete hematological remission with complete donor chimerism.

For the determination of the detailed architecture of the constitutional derivative chromosome 7, we performed chromosomal microarray and metaphase fluorescent in situ hybridization (FISH) analysis^6^. Genomic DNA and metaphases were obtained from her peripheral blood at age 2 months after obtaining written informed consent from her parents. All procedures were reviewed and approved by the institutional review board of Shinshu University School of Medicine and were in accordance with the ethical standards of the Declaration of Helsinki. We used a whole-genome oligonucleotide based-array platform consisting of 180K oligonucleotides (CGX^TM^SNP v1.1; PerkinElmer Inc., Waltham, MA). The data were analyzed with Genoglyphix software (PerkinElmer Inc.) according to the human genome assembly Feb 2009 (GRCh 37/hg19). A 26.74 Mb gain was shown in the 1q41-qter region (Fig. 1f), with an average log2 ratio of 0.527, compatible with the full trisomy state in this region. No copy number loss was detected in the 7p22.3-pter region with microarray using our platform (Fig. 1f). Signals of the 7p subtelomeric probe through metaphase FISH analysis were detected not only on the normal chromosome 7pter but also on the derivative chromosome 7p (Fig. 1g). She was concluded to have pure duplication 1q41-qter, derived from an unbalanced translocation between 1q41 and 7p22.3. The final karyotype was described as 46,XX,der(7)(1qter->1q41::7pter->7qter).

Reviewing four cases with constitutional pure duplication 1q41-qter^2, 3^ including the proband, common clinical findings observed in two or more cases included developmental delay with intellectual disability, craniofacial features (macrocephaly, prominent forehead, hypertelorism, triangle face, downslanting palpebral fissures, broad nasal bridge, micro/retrognathia, low-set ears, short neck), foot malformation, hypertrichosis, and respiratory insufficiency (Table 1), which could represent a recognizable clinical entity.

Clonal chromosomal abnormalities are detected in approximately 50% of patients of primary MDS^7^. The most frequent abnormalities are interstitial deletions of the long arm of chromosome 5 (del(5q) or 5q−) with or without additional karyotypic abnormalities, recognized as a distinct entity named 5q− syndrome according to the WHO classification^4, 5^. A large database from Austria, in which 1080 among 2072 patients with MDS (52%) were found to have clonal chromosomal abnormalities, identified deletions of 5q (30% of the 1080 patients), −7/del(7q) (21%), +8 (16%), −18/18q− (7%), 20q− (7%), −5 (6%), −Y (5%), −17/17p− (including isochromosome (17q)) (5%), +Mar (5%), +21 (4%), inv/t(3q) (4%), −13/13q− (4%), +1/1q+(3%), −21 (3%), +11 (3%), 12p−(2%), t(5q) (2%), 11q− (2%), and t(7q) (2%)^7^. Interestingly, a multi-center study from Korea, in which 92 among 205 patients (45%) were found to have clonal chromosomal abnormalities, identified trisomy 1q as the second most frequent chromosomal abnormality (15%), with trisomy 8 as the most frequent (20%)^8^. The pathogenesis of MDS associated with clonal trisomy 1q remains unknown. It is usually present with additional common abnormalities such as trisomy 8, monosomy 5, or monosomy 7, suggesting that 1q abnormalities are mostly secondary events representing clonal evolution^9^. Contrarily, the involvement of 1q heterochromatin associated with unbalanced centromeric translocation was found as the primary clonal abnormality in several patients with MDS, which suggested the importance of 1q heterochromatin in the development of MDS^9, 10^.

A 26.74-Mb gain in the 1q41-qter region shown in the proband included 95 genes listed in Online Mendelian Inheritance in Man^®^ (Supplementary Table 1). Among the 95 genes, *PARP1*, *AKT3*, and *TP53BP2* are known as oncogenes, though they have not been reported to be associated with the development of MDS^11, 12^. The abnormal expression of these genes related to oncogenesis has been reported in various human tumors, including hematopoietic malignancies^13–16^. However, there have been no reports of patients with constitutional 1q duplication who developed MDS or other hematological malignancies. Therefore, MDS in the proband might be a coincidental event and may be associated with other genetic and/or environmental factor(s).

In conclusion, we describe the detailed hematological and molecular cytogenetic findings of the fourth patient with the constitutional pure duplication 1q41-qter and the first who developed MDS among those with the constitutional 1q duplication. Common clinical features in constitutional pure duplication 1q41-qter include developmental delay, craniofacial characteristics, foot malformation, hypertrichosis, and respiratory insufficiency. The association between MDS and duplicated genes in the 1q41-qter region in the proband remains unknown.

## Electronic supplementary material

Supplementary Table 1: OMIM genes in the 27.64-Mb duplicated region at 1q41-qter in the present patient
